# The Lesson of the Manchester System

**Published:** 1913-10-04

**Authors:** 


### THE NATIONAL MEDICAL GUILD.

President: CHAS. BUTTAR, M.A., M.D., B.C.

Gen. Sec.: P. C. RAIMENT, M.R.C.S., L.R.C.P.

Offices: 34 Villiers Street, Strand, London, W.C.

### THE LESSON OF THE MANCHESTER SYSTEM.

I hope that all the readers of these lines are members of the British Medical Association, because by virtue of their membership they enjoy the privilege of reading the *British Medical Journal* every week, and I know of no more interesting way of spending an hour than to take the Supplement and to give a careful study to the correspondence, and to the report of the work of the local medical committees in various parts of the country. I say interesting, possibly melancholy would be a more correct adjective to use, for every line breathes of the discontent that I prophesied that the panel would bring, and, moreover, every line shouts out the divided opinions of the medical profession at the present moment. I presume that this work is fairly typical as a mirror of what is happening in the country, so let us turn to the reports first and see what we find.

Manchester is the first town dealt with, and under that heading we find a report of the first six months' work done under the Insurance Act. In Manchester, as everyone is aware, they have adopted a system of payment for work done, and the result is set out below.

From January 15 to March 31, the bills sent in by the local profession amounted to £34,413 8s. and payment was made at the rate of 13s. 4d. in the £. From April 1 to June 30 the bills amounted to £40,599, and payment on those amounted to 10s. in the £. The report ends: "The work done per patient per month averaged above 7s., while the money provided by the Chancellor of the Exchequer averaged about 4s., and as the number of patients could not be reduced, practitioners were still faced with the alternatives of reducing the work done or definitely accepting the rôle of philanthropists by order of the Government."

---

This should make good reading for the medical profession of Manchester, and I venture to think that if any other profession, trade, occupation, or calling were treated in this manner by this or any other Government it would not be necessary to go to the columns of their own journal to hear of it. "Philanthropists by order of the Government." What a title! Let the Government that preached of Chinese slavery and sweating put into practice a little of that human kindness that pours from them when an appeal to the country is imminent. As the Roman Emperors of old dragged their captives in chains behind them, so the Chancellor of the Exchequer binds the whole profession so that he may pose as a philanthropist at their expense.

---

After all, the importance to the profession lies not in the sympathy of the Government, the public, or anyone else, but whether the men of Manchester are satisfied that their fees should receive an abatement of 33, or 50 per cent. If they are, then it matters not; but if, on the other hand, they feel that they are being unjustly treated, are they not prepared to strike a blow for freedom from this travesty of payment for work done? Where, now, is the Plender Report, which showed with all the inviolable sanctity of figures that the profession never received more than 4s. 11d. per head per patient, and that taking into consideration all the private patients that did not help themselves to a discount of 50 per cent.? What is the value of the Chancellor's statement that there would be no bad debts, and what practice ever made 50 per cent. bad debts and survived to tell the tale? Gone! gone into the limbo of forgotten things to keep company with the rosy promises of the Chancellor of the Exchequer, for the fulfilment of which the profession is waiting, and will continue to wait.

---

So much for Manchester! The next place worthy of comment is Denbighshire. All is not well in Denbigh judging by the report of the meeting. In the first place it was reported that a medical man had refused to attend a migrant presenting a "green voucher." The clerk informed the practitioner that unless he did so they would withhold certain payments due to him.

Really there seems no limit to which Insurance Committees will go in order to obtain treatment for the insured. I am not sure whether the threat is legal or not, but to the lay mind it looks perilously like blackmail of a mild order. After all, though, they are only carrying on the methods inaugurated by the Chancellor, and so long as we continue a series of separate units, so long will these methods be successful. I am pleased to record that the local profession upheld the action of the practitioner concerned, and I shall hope to see that they will stick to their guns, undismayed by the blackleg bogey that has been used with such terrible effect in the past.

---

During the passage of the Bill through the House of Commons I had occasion to draw attention in the columns of the press to the danger to which the profession were exposing themselves by allowing the establishment of these Insurance Committees, and thereby decentralising the fight, and converting a collective bargain and a straight issue into a hundred petty squabbles, in each of which the profession would be sure to be defeated. It is with mingled feelings that I find my prophecy coming true, and I fear that the truth will become more and more manifest as time goes on, and the Committees learn what a disintegrated body we really are. The only solution seems to be to let each insurance area be an entity in itself, and to endeavour to inculcate sufficient *esprit de corps* in the profession to allow of one area to stand by another in time of trouble.

This again brings the Guild into the field of medical organisations, because of the ability that the members have of repudiating their contracts at a moment's notice if it were necessary. This, of course, means the hated strike, but I do ask the profession whether there is not a point at which their endurance will break down, and they will refuse to go on with the work. Suppose such a contretemps took place on February 1 in any year. Do they realise that they are helpless until the commencement of the next year before they can make any effective protest? These irritating pin pricks will go on until some row will happen of sufficient magnitude to justify a general strike, and on that day the profession will find that they have rejected the only means open to them to enable them to declare it.

---

The *Journal* furnishes us with another reason for our existence. In a letter written by Dr. Ward, the late assistant secretary to this Guild, there is a suggestion that a list of "blacklegs" should be kept at the offices of the B.M.A. in order that any district troubled by the noxious "whole-timer" should be able to collect some information of his past career. Further, he goes on to say, in order to guard against the risks of libel actions from the work falling into unauthorised hands, the records in question might be kept at the house of one of the officials.

I may say that for some time past the Guild has been collecting and tabulating a fairly comprehensive list of whole-timers and their past careers, and it is at the service of our members. At the same time we have no fear either in collecting, keeping, or dispensing the information because we are protected by the Trade Union Act. There was a time when Dr. Ward professed to be an admirer of those Acts which give us these immunities, now he seems more concerned to find a method by which the B.M.A. can take advantage of them without going through the formality of registering as a union. The suggestion is not a bad one, but the spectacle of Dr. Cox panting back to the office because he had forgotten the "black list" is too funny for words, and if it is as dangerous as all that then the B.M.A. had better send over to us when they require any libellous information.

---

The Non-Panel League.

The past week has been fraught with interest to the profession. There has been a recrudescence of the public interest in the resignation of a division of the B.M.A. and the founding of the Non-Panel League. What the upshot of it all will be is uncertain. That there will be no wholesale resignations from the Association is undoubted, and, indeed, I do not think it to be a desirable thing that there should be, unless some fresh organisation is in a position to take the place of the old one. The only regret that the Guild has, is that the members do not support the principles that we have endeavoured to teach, and join the trade union in such numbers that the Association would be forced to adopt the theory that we stand for, and so bring all the profession into a union.

What the Non-Panel League stands for I know not, nor can any of the officials enlighten me. Of course combination of any interest is a sound principle, and where two men hold the same views, such is human nature, that they will always found a club or society to expound those views, but of what practical use they can be I am not certain. One thing they must learn, and that is, that we have the panel, and I am convinced that we never can regain the position that we held anterior to the debacle of last January. Therefore, so far as the past is concerned they have nothing to hope for. As regards the future they may, of course, attempt to resist any fresh attacks on the profession, or any extension of the present wretched system of treating insured patients, and so far as their organisation intends to do that well and good. At the same time they are doomed to failure unless they can strengthen their numbers by the inclusion of that class of panel man who hated and still hates the Insurance Act, none the less fervently because he has the misfortune to reside in districts where the panel or nothing is the only practice open to him. The progress of the non-panel federation I shall note with interest.

---

The Guild has not altogether escaped the epidemic of resignations, and there seems a tendency for the profession to form little caves of twos and threes which of course lead nowhere. Any student of political history will tell you that the detachment of isolated groups has never been a political success. *En passant*, the reasons given for resigning are curious, and show a remarkable lack of knowledge of the work of the Guild and of the situation. However, we are not alarmed, and shall pursue the tenor of our way secure in the certainty that one day the profession will feel the need of a union, and will come into the fold. P. C. R.

